# A Birds-Eye View of Some of the More Important Points of Medico-Dental Practice, Illustrating the Importance of Thorough Education

**Published:** 1853-10

**Authors:** Wm. A. Pease

**Affiliations:** Dayton, Ohio


*A Birds-Eye View of Some of the More Important Points of Medico-Dental Practice, Illustrating the Importance of Thorough Education.* By Dr WM. A. PEASE, Dayton, Ohio.

Medicine as a science. Its antiquity and claims for esteem. From modern investigation, a brighter era about to dawn. Science an Admirer of Labor. The Mastery of the whole Science Essential to Success. The Folly of expecting Skill from Superficial Education. An Illustration from a discovery in Astronomy. An auspicious indication of the progress of Medicine. Specialities essential to Perfection. Complete Education necessary to accurate Diagnosis. Dentistry a speciality. Its prsent condition. Mechanical Dentistry, one cause of toleration of Quackery; the low estimate placed on the preservation of the teeth, by the community another. The aims of scientific Dentists, Mal-practice to extract achiug or ulcerated teeth. A different grade of Education necessary. The pernicious effects of Artificial Teeth on the Community and the Profession. A protest against them. Give the Devil his due. Too great prevalence of Artificial Teeth and undue importance attached to them in high life. The magnitude of the blessing a great curse. The criminality of ignorance in the healing art.

Medicine, as a science, presents a vast, and yet, a diversified field, wherein the most perfectly developed, and the highest cultivated of human intellects, may range and find continually new matter for investigation, new materials to analyze, and laws, hitherto unknown to our philosophy, by which the complicated machinery of our animal and mental existence is governed and adjusted with a nice equilibrium an ever varying scale, to the mutations, contingencies and changing conditions of the outward world. It is not an exact science, admitting of clear and precise definitions and rules; founded on known laws of the human economy, from which, a condition being given, their action may be predicated, and another and future condition demonstrated; but it presents many exceptions and anomalies; perhaps the offspring of the artificial habits and vices of society, but which, nevertheless, serve often to perplex and confound the most sagacious and profound. While we are not insensible to the many improvements modern investigations have produced, we are proud to acknowledge, that ours is not a new science with all the excrescencies and mushroom growths of fancy and indigestion; but is characterized by the proved truthfulness, solidity and usefulness, rather than the brilliance of its acquisitions; and possesses many of the hoary attributes of age, and reaches far back to that misty and shadowy period of mythological and legendery lore; where the tangible and concrete vanish in the exuberance of the ideal, and where, it is reported to have received a portion of the divine afflatus and enjoyed the propitious smiles of the gods; thence blessed, and diffusing blessings in its course it has descended in a constantly expanding channel laden with the accumulating wisdom and experience of the wise and good of all ages, and reaches us venerable even in its acknowledged imperfections, and disposes us to love it as our cherishing mother, and defend it from all sacriligous hands, and to cry out to all the modern inventions and isms that seek to supplant it in popular favor, *procul! o procul este profani!*

Prëeminently, this is an age of improvement! Founded on the solid basis the past has furnished us, we are enabled to accomplish wonders. All around us, in every department of science or art, in the material or immaterial world, in the heavens and in the bowels of the earth, wherever eye can reach or mind can soar, quick springing from the touch of the divining rod of modern investigations, start discoveries and inventions, richer than auriferous transmutations, and more wonderful and dazzling than the fetes of necromancy and magic; and crowding on the wings of the lightning, they come to announce themselves as ready for our use, and subservient to our command. Every moment is pregnant with something new, grand or useful; and before we can turn from the labors of the present, the next demands our attention to a fairer and nobler offspring. In the midst of this general upheval and commotion, this moulding of plastic elements, and production of new materials and compounds, from newly discovered affinities and revulsions; this preternatural activity of mind, are we not to expect, that the science of medicine will progress in a corresponding ratio with everything around it; and from the constant attrition of aroused mind, the auxillaries of a better chemical, anatomical and microscopical knowledge brought to bear upon it; a brighter era is about to dawn upon us, and unfold to our view, the mysteries of the human system, the laws of life and those remedial agents; a bountiful providence ever mindful of man, has provided to meet the wants and exigencies attendant on his sojourn here. Science is a coquettish maid, an admirer of labor; and though she may smile and dally for a time with the luxurious votaries, the more easily to beguile, and at last to elude them; she will only yield and receive the embraces of him, who, after a long and constant attendance, has toned himself up to a vigorous manhood, and proved himself worthy of her smiles, and capable of appreciating and fully enjoying her unfolded charms. Without this long and painful probation—this preliminary labor—the mastery of the whole science of medicine—I do not mean the capability of repeating like a parrot, the veiled wisdom of the text books, but the mastery of the nature and laws of the disease, the education of the senses to a nice perception and capability of taking cognizance of all the minutiæ as well as the prominent phazes of disease; of diagnosing accurately, and tracing disease from its point of manifestation to its primary seat. Without this power, the capability of going out from the stifled wards and consulting nature in her freshness, purity and beauty, and wherever in her ample fields or in the broad domains of science or art, aught can be found apposite and adapted to our wants of appropriating it as a legitimate offering to the shrine of disease—without this universal education, observation and power of continuity of thought, as well as a dexterity in executing our purpose, in adapting means to ends and capability of diving beneath the supernatant obsticles, apparent anomilies and masked phases, irreconciliable to our theories, and plunging deep down in medias seas, prying into the arcana of nature; we will never be able to discover the key, that will unlock to us all her stores and make medicine, what we so much desire—a perfect science, governed by known laws. It is folly to believe, that the seventh son, or a man without education, unless possessed of the most extraordinary genius, can, by taking a few lectures, without a long subsequent study, and the removal of almost insurmountable obstacles, become, aught but an automaton, a good nurse or respectable granny. He cannot reason from the data he possesses, from the *post hoc* deduce the *propter hoc* in time to anticipate and attack disease in its incipiency; instead of allowing it to mature and fall on him with the weight of a giant. Success, in the practice of medicine, and in the development of the laws of health and disease, must be sought for among the same class of persons, and from the same means that would produce it in the other sciences and professions. We must not calculate to maintain a high position, for science or skill, from chance, or the lucky thought of a "dunder-headed Dutchman;" but must resort to, and sedulously employ the same means that produce success in them.

Leverrier, thoroughly versed in all the known laws and phenomena of the heavens, while examining the planet Herschel, discovered that it occupied a place in the planatary system, different from what astronomers had assigned it. Their calculations were exact, the planet was astray, wandering, without law, aimless. A cause there must be—within its orbit, nothing could produce it—it must be exterior. He applied himself to the task, measured the space between the calculated and real position of Herschel, calculated the force that would produce it, gave existence and form to that force, in the shape of an unknown world, and then, calculated its distance from the sun, its size and density, and period of revolution, assigned it a place in the heavens, and then, directing the telescope to be pointed to the place, the largest of the planets, was seen whirling its way around the sun, in the exact place he had calculated, acting on, and being acted upon, by the other planets, modifying and giving regularity to their movements, and rendering them determinable, harmonious, and intelligible. Here is a beautiful example of what may be expected in any science or profession, from the investigations of thoroughly educated men. The failure of Herschel to pursue the exact course and occupy the place among the stars assigned it by mathematical calculations, had been unaccountable to a man of inferior education; but it convinced Leverrier, there must be a vast force of attraction exerted upon it at that conjunction, and that force must be an unknown planet, revolving in the unexplored regions of space. He calculated its elements, assigned it a habitation among the stars, gave it a name, and made himself as familiar with its appearance, as with the face of a friend; although he had never seen it, or had cause to believe in its existence, except by the purtubations and irregular movements of the planet Herschel.

Perhaps the most aupicious indication of the progress of medicine in our day, consists in the division of the labors and duties of the profession into specialities, and assigning them to members, who by education or talent, are the best calculated to practice them with success. Genius is ever partial, and dispenses her gifts with a chary hand; and few, however comprehensive may be their genius, or untiring their labors, can hope fully to comprehend and discharge all the duties of the profession, with equal clearness and facility. Their views must necessarily be distracted by the multiplicity of objects demanding their attention, and lack that point, penetration and clearness of outline, a closer view, a more circumscribed field, and fixed attention to one point had given. But, while we advocate the division of our profession into specialities, we wish to inculcate with all the force of thought and language, the great, the indispensible requisite of a thorough grounding, a complete mastery of the whole science, and all its adjuncts and collaterals; that may either remotely or proximately be brought to bear and shed light upon it. Thus, the surgeon should be a good physician, obstetrician, occulist, &c., so far as theory and a close examination of the operations of the professions of those departments are concerned. Once a master of these, he should bend all his energies to the perfection of himself in his peculiar department. This, with the study, not only of books, but morbid anatomy, systematically, will enable him to meet all reasonable demands, and acquit himself with credit. It is this common education of the surgeon, occulist &c., that gives them a common feeling, and causes them to fraternize, by giving them a common platform, and kindred habits of thought and study. They are all members of the same noble science, though they direct their energies to the perfection of different branches.

Without this thorough mastery of the whole science of medicine, the surgeon would be but illy calculated to decide, when to trust to the vis medicatrix naturæ, or when to resort to the knife. He might mistake an innocent excrescence for a malignant growth, and cut, where the disease was but the exponent of a specific taint, and amenable to treatment. It is the mastery of the whole science, that gives power, that begets skill. Partial education produces distorted views, and an immature judgment; which by chance may be right, but is liable to be wrong and ultimate in disaster, and in a case, where life or death, health or disease, beauty or deformity must be the fruit ; begets a fearful responstbility for him, who rashly or unadvisedly assumes the prerogatives of science, or dares to interfere. Accurate diagnosis should precede all remedies, which cannot be expected from incomplete education. We all know, that lemon juice is a specific for scurvey, but, none but a physician, acquainted with the characteristics of the disease, would think of employing it, before blundering on almost every other kind of medicine.

Having thus cursorily glanced at and reviewed, in a desultory manner, some of the causes of the progress of general and special medicine, let us turn to dentistry, a young but vigorous offshoot; but one, taking cognizance of a not unimportant branch of the diseases of man, and consider its present condition, its means of development, and its future prospects. Already has it passed the embryotic stage, and presents itself as a vigorous and comely youth, virging on manhood, around whose feet, are clustered many beautiful and artistic creations; displaying much skill, and affording high augories of its future and more mature labors.

No longer will it cohabit with mere tricksters, or seek from necromancy, the caballa, or even the wonderful potency of charms for success ; but building on a knowledge of medicine, and the diseases of the human system in general, it seeks by appropriate remedies to remove disease of the teeth and mouth ; whether appearing as primary affections, or metastatic and reflected from other disased organs or lesions. It eschews the idea, that dentistry is purely mechanical—an art ; and that the skilfull stopping of the holes in the teeth, irrespective of the character of their disease, the density of their structure, the constitution and present health of the person, his habits of life, idiosyncracies and taints, will be attended by the same uniform success, without constitutional or corrective treatment, that, the stopping of so many holes with putty by a carpenter, might be expected to produce. Dentistry is mechanical, inasmuch as many of its operations, at present, require the use of machinery, or instruments of mechanism, as in mounting artificial dentures; but its operations proper, are of equal importance, often as painful, and require as much skill and dexterity in manipulation, as those of the occulist or surgeon. It is the present necessity of discharging two-fold duties in our profession, or the junction of an art or trade, requiring little or no mechanical knowledge, and one that can be as successfully discharged by a mere artist, as by the ablest professor, and is, strictly speaking, as foreign to it, as the manufacturer of artificial legs, is to the surgeon, or eyes to the occulist; together with the general exemption of errors in practice, from a fatal termination, that has given license to men, uneducated in medicine, to attempt its practice. They know the low estimate placed on the preservation of the teeth by the community, and for what slight pretexts they will consent to loose them; and they also know, that the teeth, they cannot temporarily patch up, they can extract and replace, and they boldly venture out, when they would not have the hardihood to meddle with the tunics of the eye, the patient on the operating table, or to approach the parturient bed. From great exigencies or demands for education or high artistic skill, is begotten the skill to satisfy the demand. So long as the community are satisfied with wearing artificial teeth, and are willing to loose their own on so trifling pretexts as at present, they will be supplied with men just competent to discharge those duties and no more. Educated men are more or less gregarious, and choose those walks where they can enjoy a community of thought, a noble emulation, and just appreciation. As their education and aims are high, they require like associates; and have generally too just an appreciation of the value of their services, to consent to jostle in the crowd for pence; when in other professions, pounds are the reward. They are not satisfied with the present, and though conservative, they wish also to be progressive, and aim at a more perfect state of society, greater individual enjoyment, and a pleasure flowing from a highly cultivated understanding, and the exhibition of noble deeds. As dentists, they would seek to allay pain, discover its origin, and remove the cause that produced it, and restore the harmony and equilibrium of the system; rather than to discover the means of mutilation, and most effectively of destroying the offending organ. If they are unable to accomplish this, and are forced to resort to the knife or forceps, they feel humbled by the reflection, that their skill was not sufficient, to save and satisfy the confidence society reposes in them. Guthrie, in a recent lecture on surgery, has so fully expressed my views in regard to operations, that I take pleasure in presenting them. He says, the "amputation of a limb, is the last resource, and the opprobrium of surgery, as death is of the practice of physic. It being, nothwithstanding, impossible to do impossibilities, and save a limb or life which can no longer be preserved. art and science at that point, cease to be useful."

The practice of dentistry is not now, what it has been, or was in earlier periods of medical history, when disease and pain in the teeth, forced medical men to pay some attention to them, and employ such means as emergency demanded, or was dictated by their knowledge or caprice. Happily, much of the obscurity with which the nature of their diseases and their appropriate treatment, formerly was enveloped, has been removed, and we are able to mitigate, if not remove, most of the suffering resulting from decay, and often to restore the teeth to their healthy functions. Instead of being a field devoted to empyricism, dentistry is now frequently practiced by gentlemen of talent and cultivated minds, who bring to the study of disease, and the means suitable to prevent or arrest it, a vast fund of medical and pathological knowledge, and a large experience in morbid anatomy. Already have their labors been productive of fruits. No longer is an aching or diseased tooth (in many parts of our country), turned over to the tender mercies of the key; but a more accurate diagnosis has referred the locus morbi, often to other distant organs, or so divested disease of its terrors, as to make it obedient to prophylactic or local remedies. Formerly, the extraction of a diseased tooth was excusable, if not necessary; but with the abundant resources now at command, for quickly, and often radically curing tooth-ache, the extraction of a tooth, simply because it aches, cannot be justified. It would be as amenable to the censure of mal-practice to do so, as to amputate a limb for simple fracture, or a non-malignant tumor. The teeth are a part of the system, partaking of its idiosyncracies and taints, governed by its laws, and largely endowed with sympathy for its various organs, and responding to correctives of its morhid secretions, erethisms and taints. Disease of the same character and stage, in different individuals, will require different treatment to be successful. What would be perfectly appropriate in a billious or sanguine temperament, would be pernicious, in a scorbutic or ulcerative diathesis; and yet, both can be treated with nearly equal success. Heroic treatment might be admissible, or even necessary in one, while the most gentle would be necessary in the other. The recuperative powers of nature are ever various, and must be estimated in all difficult cases. I speak with reference to the various diseases of the mouth and dental periosteum, by which, teeth otherwise healthy, or still within the reach of operatic dentistry, are lost, or rendered comparatively valueless, by chronic inflammations or suppurations. For I hold, that comparitively few teeth, even those, where from culpable negligence, caries has been allowed to progress so far, as to undermine their structure, and leave nothing but a shell, if still sufficiently strong to sustain the pressure, necessary to consolidate a plug, can be rendered valuable for years, if not for life.

I go further, and assert as my firm conviction, founded on a practice running through a considerable number of years, in which, this part of dental practice has received attention, that these diseases of the envesting membrane of the teeth yield as readily as the same class of diseases in other parts of the system ; and though often obstinate and tenacious of their hold, the same patience, the same resort to medical skill, the same judicial application of medical means that would be applied to a diseased finger, would produce as salutary results in the restoration of the teeth to health ; and farther, that the extraction of a tooth simply because it has ulcerated, without a thorough resort to the means suitable to remove it, should cover the dentist with as much opprobrium as it would the surgeon to amputate a finger for an uncomplicated felon. But before this can be fully accomplished, before these teeth shall receive the full share of attention the importance of their preservation demands, before well directed efforts for their preservation shall become universal and be considered an essential, a ligitimate field for the dentist, much will remain to be accomplished, mountains of prejudice and ignorance will have to be removed—an educated class of dentists will have to take the place of the pullers and drawers—the more mechanical—the manufacturers of tinsel shining plates, and pretty little white teeth—the perfectors of mechanical dentistry. The people will have to be educated, the practitioners of medicine will have to be educated—educated up to that point where they will give the teeth their true and natural importance, as a part of the physical man ; and view them as living necessary organs to his perfect animal and mental developement, and not as mere parasytes, bony excresences, or accessories of beauty. They will have to be taught the relative value of natural and artificial teeth, as a means of preserving health and prolonging life, and the vast superiority in this respect of poor natural teeth to the best of the glistening, preternaturely white and small artificial ones. They will have to learn that in neglecting their teeth they are impairing their health, shortening life, lessening its enjoyments and transmitting to their children weak constitutions and teeth too delicately constituted to long resist disease till the genus homo shall become dentated ; and though art may measurably, so far as external appearance is concerned, supply the place of the natural or gans, they are rendering their mouths tasteless, foul and malarious to themselves, and their breath as repulsive to their friends as the emenations of the charnel-house. Besides, they are emasculating manhood, removing and obliterating those external signs the Creator has placed on the face as a key to the soul that burns within. They are leveling down to one common mould, and one common monotony, giving a similar expression to the giant and the dwarf, the lion, and the lamb, and clothing man with a borrowed and fiictitious and libilous expression, destroying his identity, and rendering the "human face divine as expressionless as the sickly figures in a French fashion plate.

Moreover the community must learn to distinguish the true, from the false dentist, and that great mechanical ability, although a useful accompaniment, is not a necessary qualification of a dentist; that dentistry has something higher and nobler for its aim, requiring greater talents, study and preparation, than to become a skillful dabler in pottery, or a former and swedger of dies, inasmuch as it has to deal with living, sensitive organs and tissues, to study disease in all its stages, from its insipiancy to its culmination and final termination in death; and in those forms, which more particularly fall to its province, by the skillful application of remedies, prevent or mitigate pain and restore diseased organs to their normal condition; and that it has no sympathy with the vandals who seek to destroy the handi-work of the Creator, and substitu te it is true, a beautiful, but frail ephermeral and cumbersome structure. It is the fatal facility of manufacturing artificial teeth, and the confessed ignorance of the cause of premature decay of the natural ones, as well as a want of a definite and successful method of combatting certain forms of disease that has led us all, like an *ignus fatuus*, astray, wandering through the swamps and quagmires to us, of mechanism, seeking for poisons, calcareous or silicious earths, and even invading the sooty and scathing regions of Vulcans, and compelling the service of his fires and hammers rather than high up on the mountains in a clear way, in a bracing and balmy air, with buoyant spirits, pursuing our investigations towards the *fons et origio mali*, and gathering from its bright sunny slopes, or by the margin of the clear crystal springs, those remedies, a bountiful providence has designed as a specific for every taint, a remedy for every ill. While I feel reluctant to breathe a word, to utter a sylable, to entertain an idea that could, in the remotest degree, convey an impression that I undervalue the importance of the mechanical department, or that I do not heartily approve of the labors of those who have contributed so much to the perfection of this branch and are seeking still farther to improve it; still, I must be permitted to record my dissent, to enter a protest against making it a measure of skill —the highth of dental attainment; especially to those entering the profession, to the neglect or detriment of that equally high, noble and more legitimate field of dental investigation, the study of the pathology and the therapeutic and operatic treatment of the teeth, with reference to their preservation and health. That the insertion of artificial teeth, and the perfection and facility with which it is accomplished has done much towards making dentistry what it is, by calling the attention of the community to it, and creating a confidence in its resources and utility; besides familiarizing them with the various operations on the teeth, and inducing them to look to the dentist rather than the physician, for relief, is freely admitted; still it is but reasonable to suppose that, had the same labor and talent been directed to investigating the nature of decay, and of the appropriate remedies for its prevention and cure, greater light had been thrown on these occult workings of disease, and some preventive or panacea had been produced to stay the ravages of this foul destroyer of our mouths. How gratifying soever to our feelings as professional men may be the favor with which the public receive any new improvement in mechanical dentistry, giving evidence as it does of the appreciation of the skill and labor that has been applied to this department, and has already brought it to so great perfection, both in appearance and utility; yet, in the magnitude of the blessing, the universality of its application, its capability of increasing the beauty, especially of females, and its prevention of a posibility of pain by removing the cause, there is much to arrest the attention and arouse the fears of the more calm and considerate, lest this great, this almost inestimable boon we have in our power to bestow on the afflicted humanity, by misdirection, over estimation, possibly the cupidity of some, or by diverting scientific investigation from the higher and more legitimate field of operation, instead of the great blessing it is when judiciously employed, as the dernier resort, the forlorn hope of the toothless, may become shorn of its capability of good, of its intrinsic merit, and ultimate in a great, an unmitigated evil. Already the too great prevalence of artificial teeth in the community, especially in the higher circles, and the facility with which they are inserted, has begotten an over estimation of their value, and a corresponding disregard to the natural ones. We have improved and refined the improvements in extracting instruments to such a degree that we have removed the last bulwark to the safety of our mouths. For slight and insufficient reasons ladies and gentlemen, of otherwise good information and judgment, present themselves to their dentist and desire the removal of their teeth; often when but slightly diseased, perhaps irregular or discolored, but which, by judicious treatment, might be made healthy and useful for years, if not for life. Does an erratic pain course along the cheek or head, the result of an exposure to a current of air—of an overloaded dyspeptic stomach, or an overtaxed abused nervous system,—straightway they fly to a dentist, and the poor teeth have to suffer. On many points of dental practice there seems to be no definite rules to which to refer in difficult or doubtful cases; but each one follows the bent of his genius or inclination and operates or not, as caprice or judgment dictates. This is all wrong, and should not be, as it begets contentions and disputes, lessens our fraternal feelings—our confidence in each other, and the confidence the public should repose in us. Definite land marks should be established for our government, beyond which, if an operator goes without the most urgent and obvious reasons, he should be held up to the opprobrium of an insulted profession and injured community. There being no universally recognized and published rules for the government of our profession, many are ignorant of the duties they owe to the community and the profession, with regard to operations, and often perhaps think they are doing God and humanity service in their murderous operations. To illustrate my views on this point, I will present a case. A strong man, with robust health suddenly finds his teeth defective—a part of one has crumbled off at dinner. He has seldom known disease or pain, and trusting to an iron constitution that has sustained him thus far in life, he has never anticipated the evil day, or sought for remedies for a disease he might never have. He, indeed, knows that dentists and doctors exist, and has heard that teeth are pluged, extracted and inserted, but has no clear and definite knowledge of the nature of the operation. But his teeth ache—he is half crazed and madened with pain—something must be done, and quickly. He has never reflected that there may be different grades of skill, but assumes that all who have *signs out* must be the ones ; or, if he now attempts to discriminate, he thinks it is all mechanical, and determines that the man who has a genius for mechanics, who can whittle out the most incongruous and uncouth things from a pine stick, or can make a pocket wind mill, is just the one. Perhaps he has read in the papers of the "perfectors of mechanical dentistry," and of the "matchless beauty of continuous gums," and knowing one of that ilk, he flies to him for relief. His mouth, on examination, presents the following appearance, viz: The teeth are large, strong, of dense structure, and closely set or crowded, and nearly all of them have diseases on their approximal surfaces, which has insensibly progressed, till it has undermined their structure, endangered the nerve, and the dense enamel is liable to crush in. It is a bad case—the dentist is in a fix. His skill is mechanical—he ridicules your book-men, and never reads. But the man must be attended to. He doubts his capacity to plug them and make the plugs stick, and knows he cannot doctor the inflamed nerves into quietness and make them behave. But it wont do to confess ignorance, and loose both reputation and patient—besides a large set of teeth is a fat job and will consign all the diseased teeth in the mouth to the tomb of the Capulets, beyond any tell-tale resurrection of out coming plugs. He declares the teeth must be extracted. The patient is astonished at the decison, but having a tangible proof that all is not right, submits, and in due time the set is inserted. A year has elapsed—the man has got along as well as could be expected. He has had to bestow more care on his set, to make it endurable, than he ever did on his natural teeth. But the teeth, to which it is attached, having to sustain the labor and strain of the set as well as what naturally belonged to them begin to revolt and show signs of disease. Chronic inflammation supervenes—they are lost. A whole set takes their place. The man has lost most of the pleasures of the table, mastication is with difficulty performed, the sense of taste is impaired, the stomach over worked, while his mouth is filled with a ponderous piece of mechanism, uncomfortable and disagreable to himself, and disgusting to his friends. Let us survey the man! a practised eye can already discern indications of impaired nutrition. His once ruddy countenance begins to show symptoms of anemia, his stalwart figure has lost its firm and erect carriage, atrophy siezes his iron muscles, and his aldermanic embonpoint disappears. He drags out a pleasureless existence, and prematurely dies. This is no fancy picture, but a strern reality to be met with every day. God never made the teeth for the forceps, but designed them for use during life, and such is the economy of our system, that the loss of one begets the loss of others, or serious disturbance of their arrangements. They insensibly seek the support of the lost one, and in their endeavors to fill up the void, assume unnatural and unhealthy positions, by which in occlusion they perform an increased amount of labor, and receive an unnatural pressure, while they are less able to sustain it; or, in other words, have lost the long arm of the lever. Alveolar absorption ensues, often accompanied with periosteal inflammations which tell fearfully on the teeth. Trifling though this may seem to us, who from infancy have been accustomed to measure the loss of a tooth only by the amount of pain the extraction occasions, yet, who can say but it may tell fearfully in the diminished number of years in our lives, that it diminishes our pleasures and comforts, mars and distorts our features is certain. Or, who can say that the man who unnecessarily extracts a tooth, is not as much a murderer as he who administers a slow poison. To a sensitive man, one endowed with the nicer feelings of humanity, not wholly engrossed with self, one that can occasionally disintegrate himself from men, enter in, dwell, sympathize and incorporate himself with the great mass of humanity, flowing, surging, and edying around him, and by a sort of mesmeric sympathy, an iodized delicacy in receiving impressions, perceive their wants and sufferings—to such a man what can be more harrowing than to learn that the act—treatment he had intended for their benefit, had resulted in serious evil, irreparable injury ; and that, too, from a neglect on his part to ground and thoroughly perfect himself in those studies, and that thorough knowledge of medicine, that had enabled him to diagnose the disease and afford that aid the community had a right, ex-officio, to expect at his hands. Day by day, and week by week, as he sees the victims of his cupidity or ignorance, like ghosts stalking around him, and marks how their attenuated figures become longer and leaner, their cheeks more sunken, their eyes more hollow and unearthly, and their skinny, bony fingers grasp a stick as a frail support of their unsteady step, will not his conscience like a culprit accuse him and cry I, I, I did it. I was a fool, and took on the guize of wisdom! Here is the price for which I robbed them of their health—their teeth! Then will the regular, preternaturally white and small artificial teeth be beautiful in his eyes! Will they not chatter? Will he rejoice in the perfection of his work, the matchless beauty and brilliance of his polished plates, or see perfection in continuous gums! And then will he find relief in the exclamation,—I am a good mechanic!—a respectable granny!

